# *N*^6^-methyladenosine is required for the hypoxic stabilization of specific mRNAs

**DOI:** 10.1261/rna.061044.117

**Published:** 2017-09

**Authors:** Nate J. Fry, Brittany A. Law, Olga R. Ilkayeva, Christopher L. Holley, Kyle D. Mansfield

**Affiliations:** 1 Biochemistry and Molecular Biology Department, Brody School of Medicine, East Carolina University, Greenville, North Carolina 27834, USA; 2 Department of Medicine, Duke University Medical Center, Durham, North Carolina 27710, USA; 3 Duke Molecular Physiology Institute, Duke University, Durham, North Carolina 27701, USA

**Keywords:** *N*^6^-methyladenosine, hypoxia, mRNA stabilization, post-transcriptional regulation, methyltransferase

## Abstract

Post-transcriptional regulation of mRNA during oxygen deprivation, or hypoxia, can affect the survivability of cells. Hypoxia has been shown to increase stability of a subset of ischemia-related mRNAs, including VEGF. RNA binding proteins and miRNAs have been identified as important for post-transcriptional regulation of individual mRNAs, but corresponding mechanisms that regulate global stability are not well understood. Recently, mRNA modification by *N*^6^-methyladenosine (m^6^A) has been shown to be involved in post-transcriptional regulation processes including mRNA stability and promotion of translation, but the role of m^6^A in the hypoxia response is unknown. In this study, we investigate the effect of hypoxia on RNA modifications including m^6^A. Our results show hypoxia increases m^6^A content of poly(A)^+^ messenger RNA (mRNA), but not in total or ribosomal RNA in HEK293T cells. Using m^6^A mRNA immunoprecipitation, we identify specific hypoxia-modified mRNAs, including glucose transporter 1 (Glut1) and c-Myc, which show increased m^6^A levels under hypoxic conditions. Many of these mRNAs also exhibit increased stability, which was blocked by knockdown of m^6^A-specific methyltransferases METTL3/14. However, the increase in mRNA stability did not correlate with a change in translational efficiency or the steady-state amount of their proteins. Knockdown of METTL3/14 did reveal that m^6^A is involved in recovery of translational efficiency after hypoxic stress. Therefore, our results suggest that an increase in m^6^A mRNA during hypoxic exposure leads to post-transcriptional stabilization of specific mRNAs and contributes to the recovery of translational efficiency after hypoxic stress.

## INTRODUCTION

Hypoxia is a metabolic condition that occurs when oxygen levels are deficient in cells or tissues. This stress can occur during embryonic development, where crowding and rapid cell division causes a shortage of blood and oxygen supply (Keith and Simon 2007; Mazumdar et al. 2009). Hypoxia can also be brought on by impaired blood flow due to heart attack or stroke (Semenza 2014a; Ferdinand and Roffe 2016). Other diseases can affect oxygen delivery including sickle cell disease and low blood pressure, which create hypoxic environments within the tissues. Regardless of the origins of the hypoxic stress, cells must alter their metabolism and gene expression in ways that will increase their chance of survival, or succumb to apoptosis. The physiological response to hypoxia is initiated by the stabilization of the hypoxia-inducible factor-1α (HIF-1α) transcription factor targeting genes containing a hypoxia response element (HRE) (Wenger et al. 2005; Lokmic et al. 2012; Hong et al. 2014; Semenza 2014b). HIF-1α is also important for promoting cancer cell survival through interactions with Myc and Jun, and it is well documented that hypoxia drives tumor angiogenesis (Harris 2002; Laderoute et al. 2002; Nelson et al. 2004; Dang et al. 2008; Rankin and Giaccia 2008; Li et al. 2009; Foster et al. 2014). HIF-1α directly stimulates the transcription of vascular endothelial growth factor (VEGF). This hallmark of the hypoxic response leads to increased translation of VEGF promoting vascular growth in order to increase the blood supply to affected cells, thereby leading to increased oxygen (Levy 1998; Levy et al. 1998; Stein et al. 1995; Arcondéguy et al. 2013). The hypoxic response also aids tumor migration by up-regulating the genes that are involved in the degradation of the extracellular matrix, as well as increasing the metastatic ability of the tumor and cellular proliferation through genes such as dual specificity protein phosphatase 1 (Dusp1) and hairy and enhancer of split 1 (Hes1) (Wykoff et al. 2001; Harris 2002; Giatromanolaki et al. 2003; Rankin and Giaccia 2008; Zou et al. 2013; Balamurugan 2015; Gao et al. 2015; Shen et al. 2016). Because the hypoxic response is so important to cancer cell survival, it is critical to fully understand all mechanisms occurring during hypoxia, including post-transcriptional regulation.

Although the transcriptional response to hypoxia is well established, the post-transcriptional response to oxygen deprivation is less understood. Post-transcriptional responses often regulate mRNA splicing and stability, and the stability of individual mRNAs, such as VEGF and Glut1, is increased with hypoxic exposure (Paulding and Czyzyk-Krzeska 2000). We have recently expanded upon these studies and identified numerous mRNAs stabilized in response to oxygen and glucose deprivation, including VEGF, Myc, Hes1, Jun, and Dusp1 (Carraway et al. 2017). Specific sequences in VEGF mRNA 3′ UTR and ORF have also been discovered to contribute to the stabilization of VEGF in response to hypoxia, but this analysis has not been extended to other mRNAs (Levy 1998; Levy et al. 1997, 1998; Dibbens et al. 1999; Goldberg-Cohen et al. 2002). It has also been well documented that severe oxygen deprivation leads to inhibition of global cap-dependent translation, but post-transcriptional regulation of a subset of mRNAs allows for continued translation through a number of proposed mechanisms (Liu and Simon 2004; van den Beucken et al. 2006; Young et al. 2008; Fähling 2009a,b; Uniacke et al. 2012). The hypoxic response has also recently been implicated in global changes in alternative splicing (Hu 2014; Sena et al. 2014). Thus, it is clear that post-transcriptional regulation of mRNAs has a role in the hypoxic response, but the mechanisms involving this regulation have not all been identified.

Recently, m^6^A mRNA modification has been shown to be important for the stability and translational efficiency of mRNA (Wang et al. 2014a, 2015; Du et al. 2016; Li et al. 2017; Shi et al. 2017). m^6^A methylation is a post-transcriptional modification of mRNA occurring in the nucleus (Lee et al. 2014; Liu et al. 2014; Ping et al. 2014). The m^6^A methyltransferase complex consists of methyltransferase like-3 and -14 (METTL3 and METTL14), as well as Wilms’ tumor associating protein (WTAP) (Agarwala et al. 2012; Liu et al. 2014; Ping et al. 2014). METTL3 contains an *S*-adenosyl methionine (SAM) binding domain, and utilizes SAM as a substrate to methylate target mRNAs that contain a DRACH m^6^A consensus sequence, often found in 3′ UTR's and around stop codons (Meyer et al. 2012; Liu et al. 2014), while METTL14 lacks catalytic activity but participates in mRNA binding/targeting (Śledź and Jinek 2016; Wang et al. 2016a,b). m^6^A methylation of RNA is reversible and can be removed by alkylation repair homolog 5 (ALKBH5) or fat mass and obesity related protein (FTO) (Jia et al. 2011, 2013; Fu et al. 2013, 2014; Zheng et al. 2013; Liu and Jia 2014; Meyer and Jaffrey 2014; Xu et al. 2014a). Methylated mRNA is transported out of the nucleus and bound by RNA binding proteins, including most members of the YTH family (Dominissini et al. 2012; Wang et al. 2014a; Xu et al. 2014b). While much is known about the mechanisms of m^6^A writing and erasing, the broader consequences of RNA methylation are still being investigated. m^6^A methylation has been shown to mark mRNA for degradation, mediated by YTHDF2 transport to P bodies where degradation of mRNA occurs (Fu et al. 2014; Schwartz et al. 2014; Wang and He 2014; Wang et al. 2014a). Additionally, YTHDF1 has recently been reported to stimulate translational efficiency of m^6^A methylated mRNA, thereby increasing translational output (Wang et al. 2015).

It is known that through post-transcriptional regulation, the fate of mRNAs can change based on the changing conditions of the cell (Mansfield and Keene 2009). Post-transcriptional modifications like m^6^A may alter the fate of RNA by potentially altering secondary structure, the ability of RNA binding proteins to bind, or the position of splicing events (Karijolich et al. 2010; Liu et al. 2015; Roost et al. 2015; Theler and Allain 2015; Zhou et al. 2016). Changes at the RNA level can occur rapidly and are necessary to adapt to rapidly changing microenvironments. RNA modifications can also direct permanent changes within the cell, as is the case of m^6^A modifications that limit the pluripotency of embryonic stem cells (Wang et al. 2014b; Geula et al. 2015; Zhao and He 2015). Defects in these post-transcriptional modifications could cause problems in rapid cell response mechanisms, embryonic development, or even promote tumor growth.

m^6^A mRNA has received much attention in recent years, allowing the factors involved in m^6^A mRNA methylation to be identified. However, the importance of dynamic mRNA methylation has remained elusive. Recently, roles for m^6^A mRNA in the pluripotency of stem cells in embryonic development (Wang et al. 2014b; Geula et al. 2015; Zhao and He 2015), as well as the induction of a cancer stem cell phenotype in breast cancer cells, has been described (Aguilo et al. 2015; Zhang et al. 2016). However, the m^6^A modification may function in other responses, including adaptation to changing cellular environments. Therefore, it is quite possible that the m^6^A modification may be involved in post-transcriptional hypoxic response mechanisms. Understanding the shifting landscape of m^6^A mRNA in hypoxic cells will shed light on not only how post-transcriptional regulation is altered when oxygen is lacking, but also the extent of m^6^A post-transcriptional utilization within a cell under other dynamic conditions.

## RESULTS

### Effect of hypoxia on cellular RNA modification levels

**FIGURE 1.**
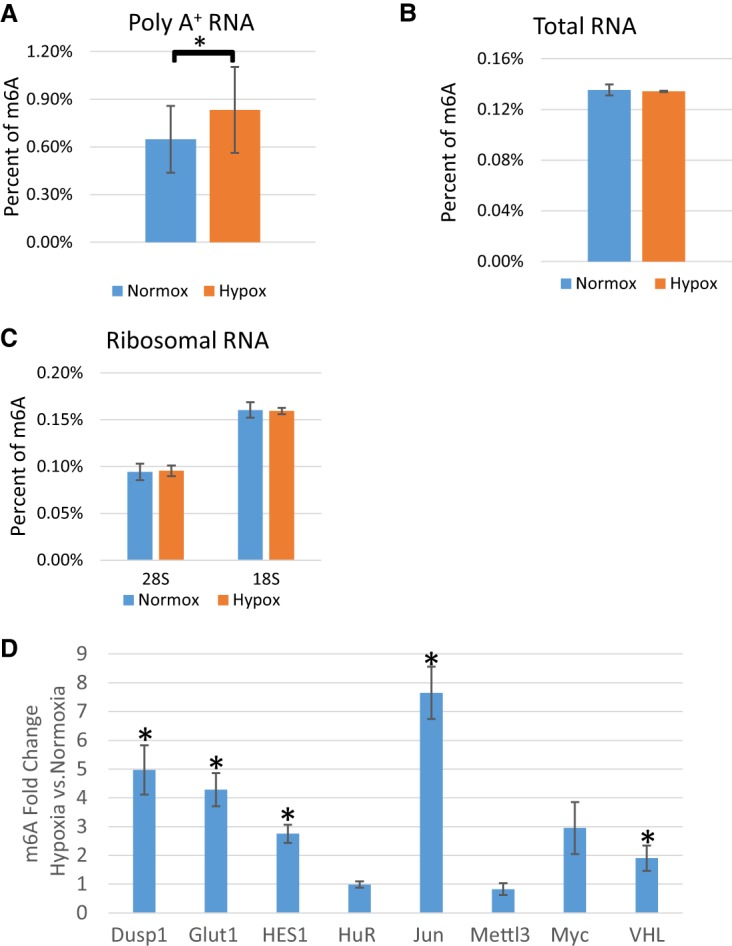
Hypoxia increases m^6^A in poly(A)^+^ RNA but not in total or ribosomal RNA. RNA isolated from HEK-293T cells grown in normoxic (Normox) or hypoxic (Hypox) conditions for 24 h. (*A*) LC-MS/MS of mRNA from HEK-293T cells. Values represent the amount of m^6^A divided by total adenosine (*N* of 3). (*) *P* ≤ 0.05 by paired Student's *t*-test. Error bars represent standard error of the mean (SEM). (*B*) LC-MS/MS of total RNA from HEK-293T cells. Values represent the amount of m^6^A divided by total adenosine (*N* of 2). (*C*) LC-MS/MS of 18 and 28S rRNA from HEK-293T cells. Values represent the amount of m^6^A divided by total adenosine (*N* of 3). (*D*) MeRIP of 100 ng of mRNA from HEK-293T cells grown in normoxic or hypoxic conditions for 24 h quantified by qRT-PCR. Fold enrichments calculated from immunoprecipitated mRNA levels normalized to input and bead-only negative control IP and expressed as a ratio of hypoxia/normoxia. (*) *P* ≤ 0.05 by paired Student's *t*-test. Error bars represent SEM of five experiments.

Changes in the m^6^A modification of mRNA have been shown to regulate stem cell pluripotency during embryonic development as well as breast cancer stem cell phenotypes. We investigated whether a cellular stress that is present in both of these models, hypoxia, has any effect on RNA modification levels, including m^6^A. To do this, HEK293T cells were incubated for 24 h under normoxic or hypoxic (1% O_2_) conditions in the presence of 1 g/L glucose to mimic the nutrient deprivation encountered during ischemic events. Poly(A)^+^ mRNA was enriched by oligo(dT) selection followed by ribosomal RNA (rRNA) depletion (verified by qPCR) (Supplemental Fig. 1A), and after fragmentation, liquid chromatography and tandem mass spectrometry (LC-MS/MS) were used to quantify various RNA modifications in the mRNA enriched samples. Surprisingly, in contrast to a recent report measuring m^6^A in total RNA and specific mRNAs in breast cancer cells (Zhang et al. 2016), global poly(A)^+^ RNA showed a significant increase in m^6^A content in HEK293T cells exposed to hypoxia (Fig. 1A). Other mRNA modifications, including 5-methylcytidine, showed no significant differences in hypoxia (Supplemental Fig. 2). These data show that the mRNA modification, m^6^A, has a dynamic response to hypoxia.

As mRNA makes up <10% of the cellular RNA mass, we postulated that hypoxia may cause changes in modifications in other RNA species as well. We tested this hypothesis by first measuring RNA modifications in total RNA. Surprisingly, in HEK293T cells m^6^A levels in total RNA were unchanged in hypoxia (Fig. 1B). However, 5-methylcytidine and *N*^1^-methylguanosine were significantly decreased (Supplemental Fig. 3). Because rRNA makes up 80%–85% of the cellular RNA mass, we postulated that modifications with diminished content in total RNA derived chiefly from changes in rRNA modifications. Thus, 40 and 60S ribosomal subunits were isolated via differential centrifugation through sucrose gradients. rRNA was isolated from the fractions and subjected to qPCR to verify the composition of the fractions (Supplemental Fig. 1B). Again, LC-MS/MS revealed no change in m^6^A content from either 18S or 28S rRNA in HEK293T cells exposed to hypoxia (Fig. 1C). However, similarly to total RNA, 5-methylcytidine exhibited a statistically significant decrease in content in 18S RNA, and *N*^1^-methylguanosine exhibited a decreasing trend in 18S RNA as well (Supplemental Fig. 4). Interestingly, pseudouridine also exhibited a decrease in content in the isolated 28S rRNA, but no changes were detected in total RNA (Supplemental Figs. 3, 4). These results suggest that not only mRNA modifications, but also specific rRNA modifications, are dynamically altered after 24 h of hypoxic conditions. However, for this study we chose to focus on the effects of hypoxia on the mRNA m^6^A content, given its reported effects on mRNA regulation.

### Identification of differentially methylated mRNAs

Given that we saw dynamic changes in the m^6^A content of mRNA, we next wanted to determine whether m^6^A methylation of specific mRNA targets involved in the adaptive response was affected when cells were exposed to hypoxia. Using m^6^A RNA immunoprecipitation (MeRIP), target mRNAs related to hypoxia and tumorigenesis including Glut1, Jun, Myc, Dusp1, and Hes1 were investigated. HEK293T cells were exposed to 24 h of normoxia or hypoxia and poly(A) mRNA isolated via oligo(dT) selection and ribominus treatment. m^6^A containing mRNAs were then immunoprecipitated using an m^6^A-specific antibody. Following cDNA synthesis, the relative enrichment of the indicated mRNAs was determined using qPCR. The targets were quantified relative to input RNA and the negative IP. Interestingly, many of the hypoxia-associated and tumorigenic mRNAs from hypoxic cells, including Glut1 and Jun, increased more than twofold in the m^6^A captured fraction, as compared to the normoxic conditions (Fig. 1D). This signifies an increase in the m^6^A content of these mRNAs in response to hypoxic exposure. It is likely that these and other mRNAs contribute to the enhanced m^6^A content of poly(A)^+^ RNA (Fig. 1A). Importantly, mRNAs such as human antigen R (HuR) and METTL3 showed no change in m^6^A content (Fig. 1D), suggesting that this was not a global phenomenon, but rather a directed change.

### Loss of m^6^A prevents hypoxic mRNA stabilization

**FIGURE 2.**
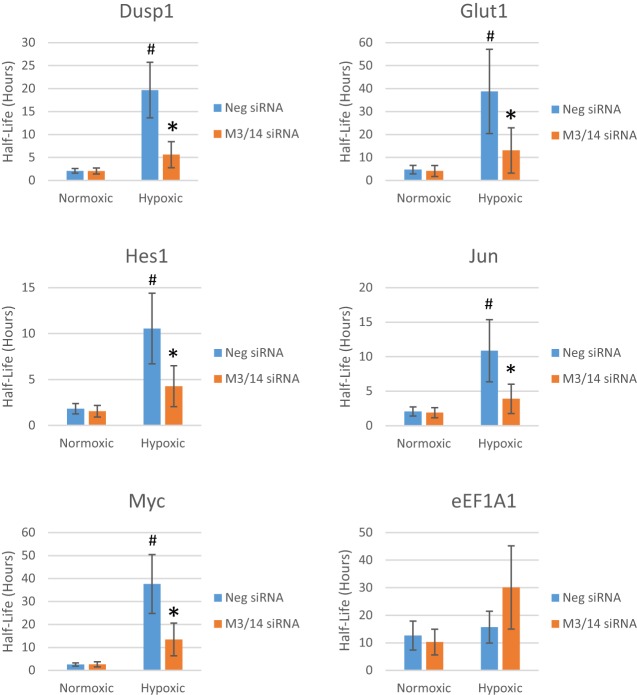
Individual mRNAs are stabilized under hypoxia in an m^6^A-dependent manner. Total RNA from HEK-293T cells was harvested after 72 h transfection with METTL3/14 siRNA (M3/14 siRNA) or negative control siRNA (Neg siRNA) and 24 h of normoxic or hypoxic conditions and half-life determined via 4sU. (#) denotes *P* ≤ 0.05 by paired Student's *t*-test between negative siRNA samples in normoxic and hypoxic conditions while (*) denotes *P* ≤ 0.05 by paired Student's *t*-test between negative and M3/14 knockdown siRNAs in the hypoxic condition. Error bars represent SEM of five experiments.

**FIGURE 3.**
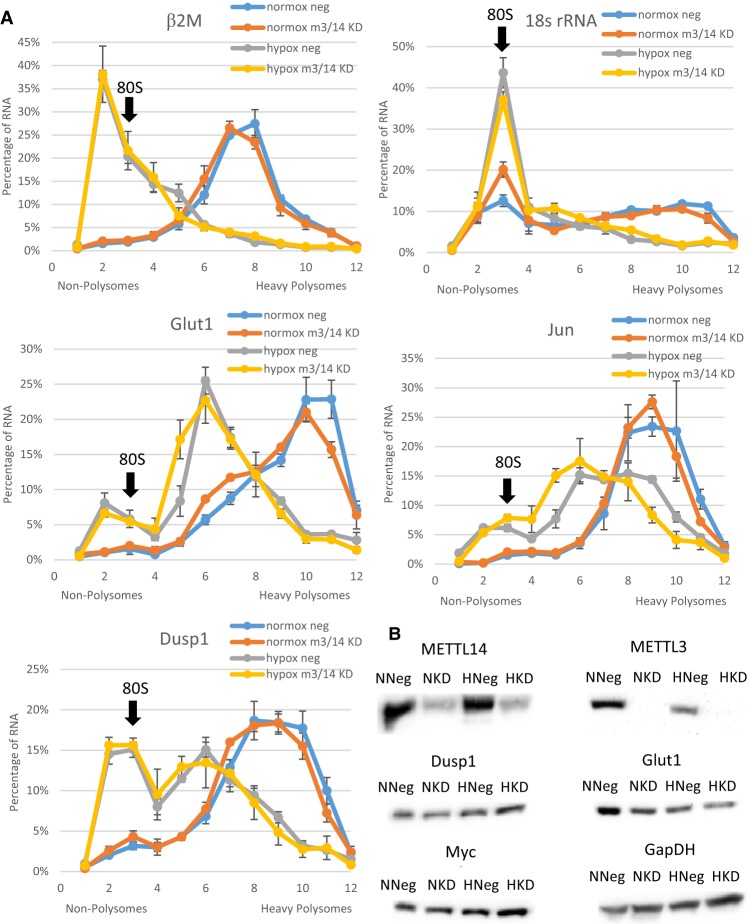
Translation rates and protein output are not affected by loss of m^6^A. HEK-293T cells harvested after 72 h transfection with METTL3/14 siRNA (M3/14 KD) or negative control siRNA (neg) and 24 h of normoxic (normox) or hypoxic (hypox) conditions. (*A*) Polysome profiling of extracts separated by differential centrifugation through sucrose gradients. qRT-PCR analysis of the fractions shows percentage of individual mRNA in each fraction. Error bars represent SEM of three experiments. Fraction containing 80S peak is marked. (*B*) Western blots of 50 µg of protein lysates of normoxic negative control siRNA (NNeg), normoxic METTL3/14 knockdown (NKD), hypoxic negative control (HNeg), hypoxic METTL3/14 knockdown (HKD) (representative of three experiments).

To address if m^6^A methylation plays a role in stabilization of these particular mRNAs as part of their post-transcriptional response to hypoxia, METTL3 and METTL14 of the m^6^A methyltransferase complex were knocked down via siRNA and the half-lives of our target mRNAs were determined. Knockdown of METTL3 and METTL14 was confirmed via Western blot analysis (Fig. 3B). Depletion of m^6^A content in individual mRNAs was confirmed by MeRIP followed by qPCR (Supplemental Fig. 5). After depletion of METTL3 and METTL14 for 48 h, HEK293T cells were transferred to either normoxic or hypoxic conditions for 24 h to simulate ischemia. During the last hour of treatment, newly transcribed RNA was metabolically labeled using 4-thiouridine (4sU). RNA was harvested, the 4sU labeled RNA was biotinylated, and streptavidin beads were used to separate the new labeled RNA from unlabeled RNA. RNA half-lives were determined from the ratio of the labeled to unlabeled RNA normalized to total RNA. As shown in Figure 2, many of the mRNAs that had increased m^6^A methylation in hypoxic conditions (Glut1, Jun, Dusp1, and Hes1) also showed a significant increase in mRNA half-life. Interestingly, knockdown of METTL3/14 had no effect on the normoxic half-life of any of the mRNAs, but loss of the methyltransferases did significantly inhibit their hypoxic stabilization. eEFA1, which exhibited no change in m^6^A level (data not shown) also showed no loss of stabilization after METTL3/14 depletion. Therefore, mRNAs that were stabilized under hypoxic conditions lost that stabilization after an engineered decrease in m^6^A levels. This suggests that enhanced m^6^A modification of specific mRNAs can indeed affect their hypoxic post-trasnscriptional regulation.

### The effect of m^6^A on mRNA translation and protein levels

Having determined that changes in m^6^A content affected mRNA stabilization, we next examined how methylation might affect the translation of these messages. HEK293T cells were again depleted of METTL3/14 for 48 h, and transferred to either normoxic or hypoxic conditions for 24 h followed by 15 min of cyclohexamide treatment and fractionation of cellular extracts on sucrose gradients. Polysome profiles (Supplemental Fig. 6A–D) were analyzed by qRT-PCR for specific mRNA targets. Sedimentation position as shown by polysome profiling allowed us to determine how efficiently each message was being translated. During sucrose-gradient resolution, mRNAs partition based on the number of ribosomes bound, which is a direct measure of their translational efficiency. For example, mRNA such as β-2-microglobulin (β2M), which is found to peak in fraction 8 in normoxic conditions, is considered to be moderately translated, while an mRNA that is primarily located in fractions 10 and 11, such as Glut1, is bound heavily by polysomes, indicating highly efficient translation (Fig. 3A). In general, hypoxic exposure caused a decrease in translational efficiency (as indicated by a leftward shift in the gradient) of assayed mRNAs. The extent of the decrease depended on the mRNA being investigated. Surprisingly, depletion of m^6^A via METTL3/14 knockdown had no effect on the polysome loading of any of the tested mRNAs, suggesting that m^6^A does not play a role in their translational efficiency (Fig. 3A).

**FIGURE 4.**
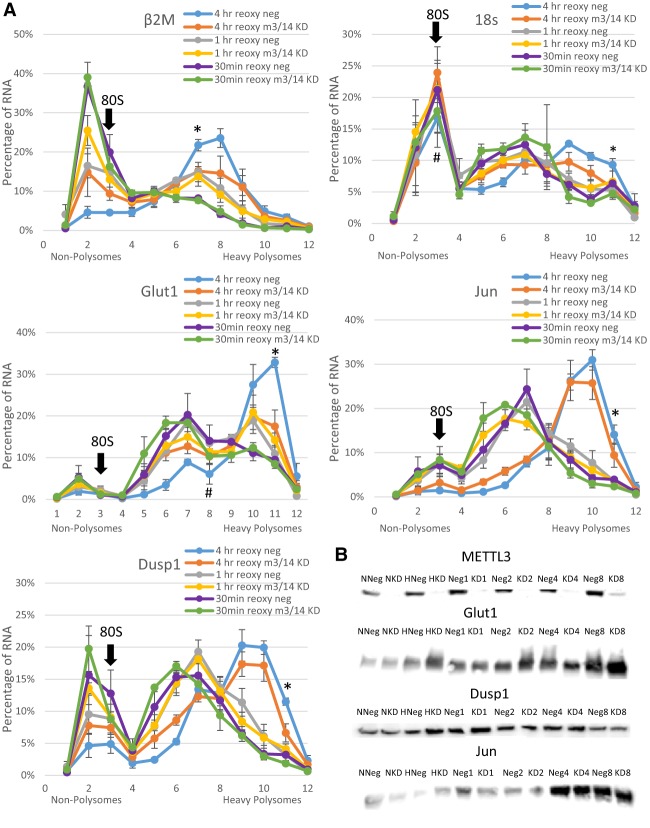
METTL3/14 Knockdown decreased the ability of messages to recover from hypoxic stress after 4 h reoxygenation. (*A*) HEK-293T cells harvested after 72 h transfection with METTL3/14 siRNA (M3/14 KD) or negative control siRNA (neg) and 24 h of hypoxic conditions and either 30 min, 1, or 4 h of room level reoxygenation recovery (reoxy). Polysome profiling of extracts separated by differential centrifugation through sucrose gradients. qRT-PCR analysis of the fractions shows percentage of individual mRNA in each fraction. Error bars represent SEM of three experiments in the 1 and 4 h reoxygenation experiments and SEM of two experiments in the 30 min reoxygenation experiment. Fraction containing the 80S peak is marked. Paired Student's *t*-test indicates significant (*P* ≤ 0.05) decrease (*) or increase (#) compared to negative control siRNA. (*B*) Western blots of 50 µg of protein lysates of normoxic negative control siRNA (NNeg), normoxic METTL3/14 Knockdown (NKD), hypoxic negative control (HNeg), hypoxic METTL3/14 Knockdown (HKD), and 1, 2, 4, or 8 h reoxygenation after hypoxia (representative of three experiments).

To determine whether increased m^6^A during hypoxia was involved in the recovery after hypoxia, polysome profiling was used to determine translational efficiency after reoxygenation of HEK293T cells with and without METTL3/14 knockdown. Polysome profiles were obtained similarly to the previous experiments with the exception that cells were exposed to room level oxygen for 0.5, 1, or 4 h prior to cyclohexamide treatment and fractionation of cellular extracts on sucrose gradients. Profiles (Supplemental Fig. 7) were again analyzed by qRT-PCR for our specific mRNA targets. After 4 h of reoxygenation, recovery of translational efficiency of Glut1, Jun, Dusp1, Hes1, and Myc was diminished by METTL3/14 knockdown compared to negative control siRNA transfected cells (Fig. 4A; Supplemental Fig. 8). These results suggest that m^6^A may be aiding the cellular response to recovery after stress.

All of the mRNAs shift from heavier to lighter fractions in cells exposed to hypoxic conditions; however, some mRNAs shifted farther into lighter fractions than others. For example, β2M and 18S shift completely out of polysomes into the subribosomal fractions 2–3. However, Glut1 and Jun shifted only moderately from heavy fractions, with the majority of the mRNA being found in fractions 5 through 7. This initially suggested that Glut1 and Jun were still being moderately translated under hypoxia as has been previously reported (Lai et al. 2016). However, it was also possible that mRNAs such as Glut1 and Jun had actually been released from the translating ribosomes but were still in large ribonucleoprotein complexes that might also increase their sedimentation in the gradient. To test whether the mRNAs were still bound by intact ribosomes and hence translating, extracts were treated with EDTA prior to sucrose sedimentation (Supplemental Fig. 6E,F). EDTA chelates the magnesium necessary for large and small ribosomal subunit association, releasing all mRNAs to presumably sediment slower in their nontranslating state. Interestingly, upon EDTA release, normoxic β2M shifted completely from heavy-to-light fractions just as observed with hypoxia with or without EDTA treatment (cf. Supplemental Fig. 9 and Fig. 3A). However, Glut1 and Jun only shifted modestly from the heavier fractions to intermediate fractions, despite being released from ribosomes. The sedimentation of these mRNAs in intermediate fractions may indicate association with previously uncharacterized ribonucleoprotein complexes which are unaffected by EDTA metal chelation.

We attempted to correlate the mRNA translational efficiency to the steady-state level of their protein products. If m^6^A increases the stability of these mRNAs under hypoxic conditions, and knocking down m^6^A decreases their stability but maintains their translation, one might expect to see a decrease in their protein after m^6^A knockdown. However, Western blotting for these proteins under normoxia and hypoxia after METTL3/14 knockdown showed no changes in protein levels of Glut1, Myc, and Dusp1 (Fig. 3B). There were also no observed changes in protein levels after reoxygenation even though polysome analysis suggested that METTL3/14 knockdown decreased translational efficiency after 4 h of recovery (Fig. 4B). This suggests that other factors likely are involved in determining the steady-state levels of proteins derived from these mRNAs. Interestingly, METTL3/14 knockdown also had no significant effect on HEK-293T cell proliferation under normoxic or hypoxic conditions (Supplemental Fig. 10).

### Summary

Our findings suggest that m^6^A mRNA methylation of certain mRNAs is induced by hypoxia. Furthermore, increased m^6^A prolongs the half-life of specific mRNA targets. Although the increased stability of these messages did not correlate with translational efficiency or changes in protein levels during hypoxia, we found that loss of m^6^A through METTL3/14 knockdown decreased the cells’ ability to recover translational efficiency after reoxygenation following hypoxic stress. We also observed that under hypoxic conditions, some mRNAs may be associated with other ribonucleoprotein complexes instead of actively translating polysomes.

## DISCUSSION

m^6^A has received renewed attention over the past few years as a dynamic mRNA modification with many potential cellular functions. Although the factors involved in m^6^A mRNA methylation have been identified, the importance of mRNA methylation remains unclear. We now suggest a role for dynamic changes in mRNA m^6^A content in regulating mRNA stability in response to oxygen deprivation. In contrast to the total and rRNA, we saw a significant increase in the m^6^A content of mRNA levels in hypoxic cells, suggesting that the m^6^A modification may be important for regulating mRNAs in hypoxia. Our LC-MS/MS results did indicate the presence of other dynamic RNA modifications in total and ribosomal RNA after 24 h of hypoxia, but we have yet to identify what the dynamic regulation of these modifications may signify.

Immunoprecipitation of m^6^A followed by qRT-PCR allowed us to determine the m^6^A methylation status of individual mRNAs. We observed an increase of m^6^A in specific mRNA targets after hypoxic exposure. However, this method cannot determine how hypoxia affects m^6^A methylation at specific sites. In follow-up studies, we will seek to determine whether hypoxia introduces m^6^A in new sites and if so, determine how specificity is regulated. It is possible that a hypoxic switch in methylation sites, for example, switching methylation from the 5′ end to the 3′ end of the mRNA, could also alter the regulation of the mRNA without affecting the overall m^6^A level.

Others have previously reported that stability of individual mRNAs was increased by hypoxia (Czyzyk-Krzeska et al. 1994; McGary et al. 1997; Levy et al. 1998; Paulding and Czyzyk-Krzeska 2002; Carraway et al. 2017). It is also known that m^6^A can affect the stability of mRNAs (Wang et al. 2014a,b). We now show that an increase in m^6^A methylation is correlated with increased stabilization of a number of mRNAs under hypoxic conditions. We confirmed the stabilization of Glut1 and Myc mRNA under hypoxia (Chen et al. 2001; Carraway et al. 2017) but also identified several novel targets including Dusp1, Hes1, and Jun. Interestingly, these findings contradict suggestions that increased methylation leads to increased degradation of mRNAs through YTHDF2 association (Zhang et al. 2016). The two data sets are not directly comparable however, as stability of those messages had not been reported under hypoxic conditions. There are a number of possible reasons for this discrepancy. Hypoxia may switch the location of the methylation, allowing for different functions of the YTH family proteins, or equally possible, an entirely different RNA binding protein may be interacting with these hypoxically methylated mRNA. We will attempt to probe these ideas in future studies.

Even though m^6^A increased the stability of certain mRNA's under hypoxic conditions, there was no noticeable effect on translational efficiency or protein level. Polysome profiling detected no substantial changes in translation between samples containing or lacking METTL3/14. Remarkably, even though mRNA stability was increased, there was no detectable change in protein levels of Glut1, Myc, and Dusp1 as determined by Western blotting. Interestingly, even though METTL3/14 knockdown did not affect translation, hypoxia itself caused a shift from heavier polysomes to a lighter complex. This shift to lighter fractions was more robust in some mRNAs than others. It was previously thought that this lack of a complete shift out of polysomes ultimately indicated the maintenance of translation. Indeed, it has previously been reported that numerous mRNAs, including Hif-1α and Glut1, continue to associate with polysomes during hypoxic conditions (Thomas and Johannes 2007; Lai et al. 2016). However, our data now suggest that these messages may be being maintained in mRNPs that do not contain 80S ribosomes, based on their resistance to EDTA-mediated disruption of ribosome association. None of the previous reports tested for this possibility.

Knockdown of METTL3/14 did decrease the ability of cells to recover translational efficiency after 4 h of reoxygenation following hypoxia. Detection of protein levels by Western blotting after reoxygenation again showed little difference with or without METTL3/14 KD, but polysome profiling showed decreases in translational efficiency after 4 h of reoxygenation in specific messages including Glut1, Hes1, Dusp1, and Myc, suggesting that m^6^A's role in hypoxia may be related to recovery after the hypoxic stress rather than adaption to the stress. It is possible that the difference between these two data sets is due to differences in protein stability, or it is also possible that we could not detect these subtle changes in protein levels accurately with Western blotting. Utilizing a more physiologically relevant cell model or investigating this phenomena in vivo might reveal situations in which m^6^A exerts a more dramatic effect in the adaptation to and recovery from hypoxic exposure.

Overall, this study demonstrates that hypoxic exposure can indeed induce changes in multiple RNA modifications. In particular, the m^6^A modification of mRNA is necessary for increased stability under hypoxic conditions. Future studies will explore these m^6^A changes with base-specific precision, as well as the RNA binding proteins that interact with the m^6^A modification under hypoxia. It is our goal to gain a better understanding of mRNA dynamics in response to hypoxia with the hope of developing new therapeutics targeting cardiovascular disease, cancer and other diseases that involve periods of reduced oxygen.

## MATERIALS AND METHODS

### Cell lines

HEK293T (HEK293T/17; CRL-11268) cells were obtained directly from ATCC and maintained in high glucose (4 g/L) DMEM (Corning/Mediatech) supplemented with 10% FBS (Atlanta Biologicals), 2 mM glutamine (Corning/Mediatech), and 1× Pen/Strep (Corning/Mediatech) and passaged when ∼85%–90% confluent. Cells were tested for mycoplasma upon receipt. For experiments, cells were plated on 10 cm dishes (CytoOne, USA Scientific) in high glucose (4.0 g/L) media and allowed to attach/recover for 18–24 h. The next day, the media were removed and replaced with media containing 1 g/L glucose. Hypoxic treatments were carried out in a Ruskin In Vivo 400 Hypoxia Hood (The Baker Company) maintained at 37°C, 5% CO_2_, 70% humidity, and 1% oxygen. All other chemical reagents were obtained from Sigma-Aldrich unless otherwise specified.

### RNA extraction

TRIzol (Life Technologies) was used for all RNA extractions according to the manufacturer's protocol. RNA was further purified and treated with RNase-Free DNase I (Life Technologies) using the PureLink RNA Mini Kit (Life Technologies). For RNA extraction from ribonucleoprotein immunoprecipitations (RNP-IP) and sucrose gradients, GlycoBlue (Life Technologies) was added as a carrier during the precipitation step. RNA quality and quantity were determined via NanoDrop 1000 (ThermoFisher Scientific).

### Poly(A)^+^ RNA purification

Poly(A)^+^ RNA was first purified from total RNA through oligo(dT) selection using a poly(A) Purist-MAG magnetic mRNA Purification Kit (Life Technologies), followed by ribosomal RNA depletion using a RiboMinus Eukaryote Kit (Life Technologies) according to the manufacturer's protocols.

### LC-MS/MS of poly(A)^+^ RNA for m^6^A modification analysis

Poly(A)^+^ RNA was hydrolyzed enzymatically by first denaturing the RNA at 95°C followed by immediate placement on ice. Poly(A)^+^ RNA was incubated with S1 nuclease buffer and 1 unit of S1 nuclease (Life Technologies) per 300 ng of RNA for 30 min at 37°C. Alkaline phosphatase buffer, 1 unit of alkaline phosphatase per 300 ng of RNA (Life Technologies), and 0.00025 units of venom phosphodiesterase I (Sigma-Aldrich) were added to incubate for 30 min at 37°C. Fragmented RNA was purified through two rounds of chloroform extraction. LC-MS/MS quantification of m^6^A and adenine was performed by Craft Technologies. Separations and identification by LC-MS/MS were performed using a Thermo Finnigan Linear Ion Trap Quadrapole (LTQ) mass spectrometer utilizing an electrospray ionization interface in selected reaction monitoring mode connected to the Agilent 1100 autosampler and Agilent 1100 HPLC pump system (Agilent). Detection was performed using an electrospray ionization source operated in positive ion mode.

### LC-MS/MS of poly(A)^+^, total, and ribosomal RNA for nucleoside modification analysis

Purified RNA was digested to individual nucleosides and modified nucleosides were quantified as previously described (Gokhale et al. 2016). Briefly, digestion was performed with nuclease P1 (Sigma, 2U) in buffer containing 25 mM NaCl and 2.5 mM ZnCl_2_ for 2 h at 37°C, followed by incubation with Antarctic Phosphatase (NEB, 5U) for an additional 2 h at 37°C. Nucleosides were then separated and quantified at the Duke Molecular Physiology Institute using UPLC-MS/MS as previously described (Basanta-Sanchez et al. 2016), except acetic acid replaced formic acid in the mobile phase.

### Ribosomal subunit separation

Cells grown in normoxic or hypoxic conditions were harvested in “Buffer A” (35 mM Tris pH 7.5, 70 mM KCL, 9 mM MgCl, 0.1 mM EDTA, 250 mM sucrose, 0.5% sodium deoxycholate, 1% Triton X-100, 1× protease inhibitors, 1 mM DTT, and RNase out). Cell lysate was centrifuged for 15 min at 15,000*g* in a Beckman TLA 100 rotor. The supernatant was removed to a new tube and centrifuged in the same rotor at 150,000*g* for 90 min. Ribosome pellets were resuspended in “Buffer B” (10 mM Tris pH 7.5, 500 mM KCL, 10 mM MgCl_2_ 1× protease inhibitors 1 mM DTT and RNase out) and layered on a 12 mL 15%–30% sucrose gradient in buffer B. The gradient was centrifuged at 86,000*g* for 14 h in a Beckman SW-41 Ti swinging bucket rotor. Of note, 1 mL fractions were collected from the top of the gradient and the positions of the 40S and 60S ribosomal subunits were found by measuring each fraction at an absorbance at 254 nm. RNA was isolated from each sample via TRIzol.

### m^6^A mRNA immunoprecipitation (MeRIP)

m^6^A ribonucleoprotein immunoprecipitation reactions were performed by first isolating poly(A)^+^ RNA from normoxic and hypoxic cells. Protein G Dynabeads (Thermo Fisher Scientific) were washed 3× in 1 mL of IPP buffer (10 mM Tris-HCL pH 7.4, 150 mM NaCl, 0.1% NP-40). Twenty-five microliters of beads were required per IP. Anti-*N*^6^-methyladenosine mouse monoclonal antibody (EMD Millipore, MABE1006) was added to the beads (5 µg/IP) and brought up to 1 mL with IPP buffer. As a negative control, beads without antibody were used as well. Bead mixture was tumbled for 16 h at 4°C. Beads were washed 5× with IPP buffer and 100 ng of poly(A)^+^ RNA was added to the beads along with 1 mM DTT and RNase out. The mixture was brought up to 500 µL with IPP buffer. Bead mixture was tumbled at 4°C for 4 h. Beads were washed 2× in IPP buffer, placed into a fresh tube, and washed 3× more in IPP buffer. m^6^A RNA was eluted off the beads by tumbling 2× with 125 µL of 25 mg/mL *N*^6^-methyladenosine-5′-monophosphate sodium salt (Chem-Impex International Inc.). Supernatant was added to TRIzol-LS followed by RNA isolation as per manufacturer's protocol. The final RNA sample was brought up in 10 µL of water.

### PCR for MeRIP

Reverse transcription was performed on 10 µL m^6^A poly(A)^+^ RNA from the MeRIP with the iScript cDNA Synthesis Kit (Bio-Rad Laboratories). After diluting cDNA twofold, quantitative real-time PCR was performed using a Roche Lightcycler 96 with Fast Start Essential DNA Green (Roche Diagnostics Corporation) and primers from Integrated DNA Technologies, Inc. Primers used are listed in Supplemental Table 1. Primer efficiency was verified to be over 95% for all primer sets used. Quantification of mRNA from the MeRIP was carried out via ΔΔCT analysis against nonimmunoprecipitated input RNA and RNA pulled down from non-antibody bound beads. All real-time PCR primer sets were designed so the products would span at least one intron (>1 kb when possible), and amplification of a single product was confirmed by agarose gel visualization and/or melting curve analysis.

### siRNA transfections

Either a negative siRNA (Silencer; Life Technologies) or METTL3 and METTL14 siRNAs (Qiagen) transfected together using a Lipofectamine RNAi Max 54 µL/plate as per manufacturer's protocol (Life Technologies) using a 180 pM siRNA/10 cm dish. siRNAs used can be found in Supplemental Table 2. Cells were incubated for 48–72 h post-transfection with the last 24 h in either normoxic or hypoxic conditions.

### 4sU

mRNA half-life determinations using 4sU were performed as per established protocol (Dölken et al. 2008). Cells were treated with 200 µM 4sU (Sigma-Aldrich) for 1 h. RNA isolated via TRIzol was biotinylated by labeling 50 µg RNA in a reaction mixture with 50 µL 10× Tris/EDTA buffer (TE), 100 µL 1 mg/mL Biotin-HPDP (EZ-Link Biotin HPDP, Thermo Scientific) in dimethylformamide (DMF), and RNase-free H_2_O brought to 400 µL. The mixture was incubated in the dark with rotation for 1.5 h. Biotinylated RNA was extracted using an equal volume of Chloroform/Isoamyl alcohol (24:1) 2× in phase lock gel heavy tubes (5 Prime) followed by RNA precipitation with isopropanol. RNA was heated to 65°C for 10 min and placed immediately on ice. RNA was added to Dynabeads MyOne Streptavidin C1 (Thermo Fisher Scientific) that had been thoroughly washed and resuspended in 2× streptavidin binding buffer (2× TE, 2 M NaCl). The RNA bead mixture was incubated with rotation for 30 min. Beads were washed 5× with 65°C wash buffer (1× TE, 1 M NaCl, 0.1% Tween20) and the supernatant was kept containing the nonlabeled RNA. Three rounds of 100 mM dithiothreitol (DTT) elution followed by one round of TE eluted labeled RNA from the beads. RNA was isolated via Isopropanol and resuspended in 40 µL of water.

### mRNA half-life

mRNA levels were determined by real-time quantitative PCR using 1 µL of RNA from both labeled and unlabeled 4sU samples. Decay rates were calculated by the natural log of one minus the RNA input normalized ratio of labeled over unlabeled RNA. All half-lives were normalized to GAPDH half-life which was defined as 8 h based on previous publications and our unpublished data (Payne et al. 2014).

### Polysome profiling

Cells were treated with 200 µM cyclohexamide for 15 min prior to harvest. Cells were harvested in PLB buffer (100 mM KCl, 5 mM MgCl_2_, 10 mM Hepes, 0.5% NP40, 200 µM cyclohexamide, 1mM DTT, 1× protease inhibitors, RNase out) and incubated on ice for 30 min. Lysates were precleared by centrifugation for 8 min at 5000*g*. Supernatant was layered on a 10%–45% sucrose gradient in polysome profile buffer (300 mM KCl, 50 mM Hepes, 10 mM MgCl_2_, 200 µM cyclohexamide). The gradient was centrifuged at 38,000*g* for 1 h 45 min in a Beckman SW-41 Ti swinging bucket rotor. Twelve 1 mL fractions were collected from the top of the gradient and the polysomes were measured from lightest to heaviest at an absorbance at 254 nm. RNA was isolated from each sample using TRIzol.

### Profiling PCR

Reverse transcription was performed on 1 µL of RNA from each fraction in a 20 µL reaction. After diluting cDNA fivefold, quantitative real-time PCR was performed. Percentages of mRNA per fraction was carried out via ΔCT analysis using a baseline *Cq* value representing 0% mRNA. Percentage of ribosomal RNA was calculated in the same manner.

### Western blots

Whole-cell lysates were prepared in whole-cell extract buffer (WCEB: 50 mM Tris pH 7.4, 150 mM NaCl, 5 mM EDTA, 0.1% SDS, and complete protease inhibitor [Promega]). Equal amounts of protein (30–50 μg) were electrophoresed on a mini-PROTEAN any KD acrylamide gel (Bio-Rad Laboratories) and transferred to Hybond ECL nitrocellulose (GE Healthcare). The transfer was verified via Ponceau S staining, then the blot was blocked with 5% nonfat dry milk (LabScientific) in Tris-buffered saline with 0.1% Tween 20 (TBST) for 1 h at room temperature, followed by primary antibody in blocking buffer overnight at 4°C. After washing extensively with TBST, blots were incubated for 1–2 h at room temperature with appropriate HRP-linked secondary antibody (GE Healthcare), washed again with TBST, developed using Pierce ECL Western Blotting Substrate (ThermoFisher Scientific), and exposed to film for detection. Primary Antibodies used and their concentrations can be found in Supplemental Table 3.

### Statistical analysis

All experiments were performed on at least three separate occasions to generate biological replicates unless otherwise indicated. qPCR was performed at least twice on each cDNA for technical verification of data. Half-lives were calculated for each biological replicate and then averaged together to determine final value and standard error of the mean. Statistical significance was calculated by a two-tailed, paired Student's *t*-test comparing experimental to control conditions. A *P*-value below 0.05 was defined as statistically significant.

## SUPPLEMENTAL MATERIAL

Supplemental material is available for this article.

## Supplementary Material

Supplemental Material
