# Supplementary material for: N6-methyladenosine is required for the hypoxic stabilization of specific mRNAs
Source: RNA. 2017 Sep;23(9):1444–55. doi: 10.1261/rna.061044.117 (PMC5558913; doi:10.1261/rna.061044.117)
Supplement: Supplemental Material [file supp_23_9_1444__index.html]

N6-methyladenosine is required for the hypoxic stabilization of specific mRNAs — Supplemental Material 

# *N*6-methyladenosine is required for the hypoxic stabilization of specific mRNAs

## Supplemental Material

- Supplemental\_Material.pdf
