# Supplementary material for: N6-methyladenosine is required for the hypoxic stabilization of specific mRNAs
Source: RNA. 2017 Sep;23(9):1444–55. doi: 10.1261/rna.061044.117 (PMC5558913; doi:10.1261/rna.061044.117)
Supplement: Supplemental Material [file supp_061044.117_Supplemental_Material.pdf]

Supplemental Figure 1

A.

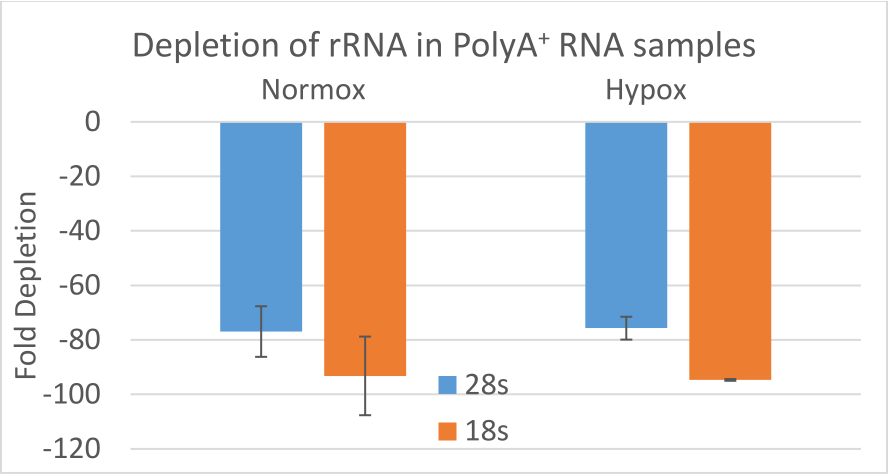

B.

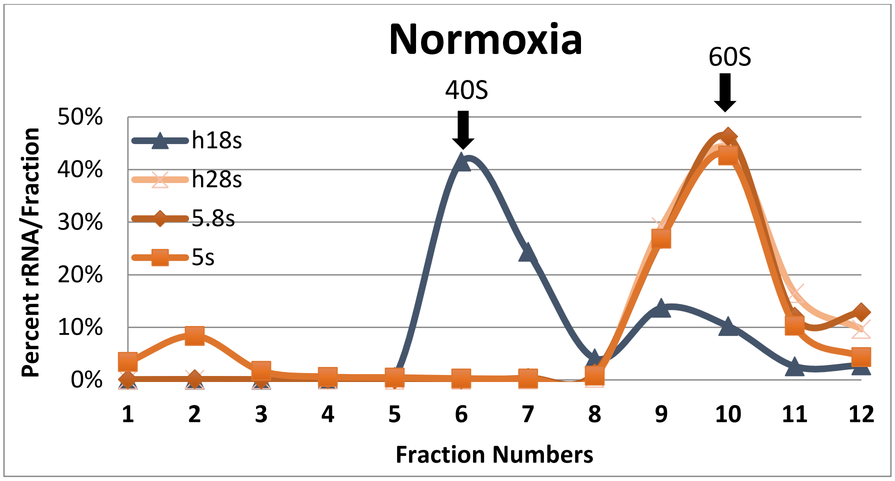

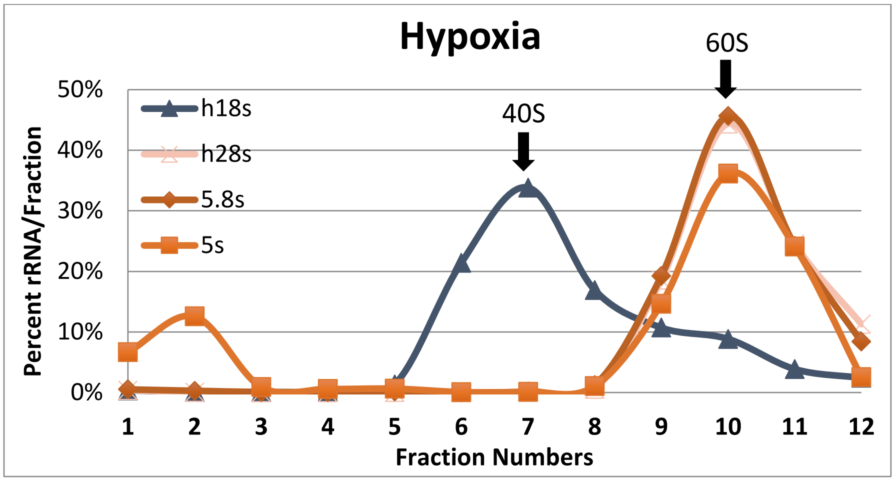

Supplemental Figure 1:

(A) qRT-PCR verification of rRNA depletion in PolyA+ RNA. Both 18 and 28s rRNA from cells cultured in Normoxic or 24 hours Hypoxic conditions were depleted over 70-fold indicating greater than 98% depletion of rRNA. (N of 2)

(B)

40 and 60S ribosomal subunits from HEK-293T cells grown in normoxic or hypoxic conditions for 24 hours were separated by differential centrifugation followed by sucrose gradient fractionation. qRT-PCR of individual fractions reveals purity of respective rRNAs in each fraction. Fractions containing 40S and 60S peaks are marked. Fractions with highest purity were used in immunoblot in Figure 1D. (Graph represents 1 of 3 experiments).

Supplemental Figure 2 **UPLC-MS/MS of PolyA$^+$ RNA**

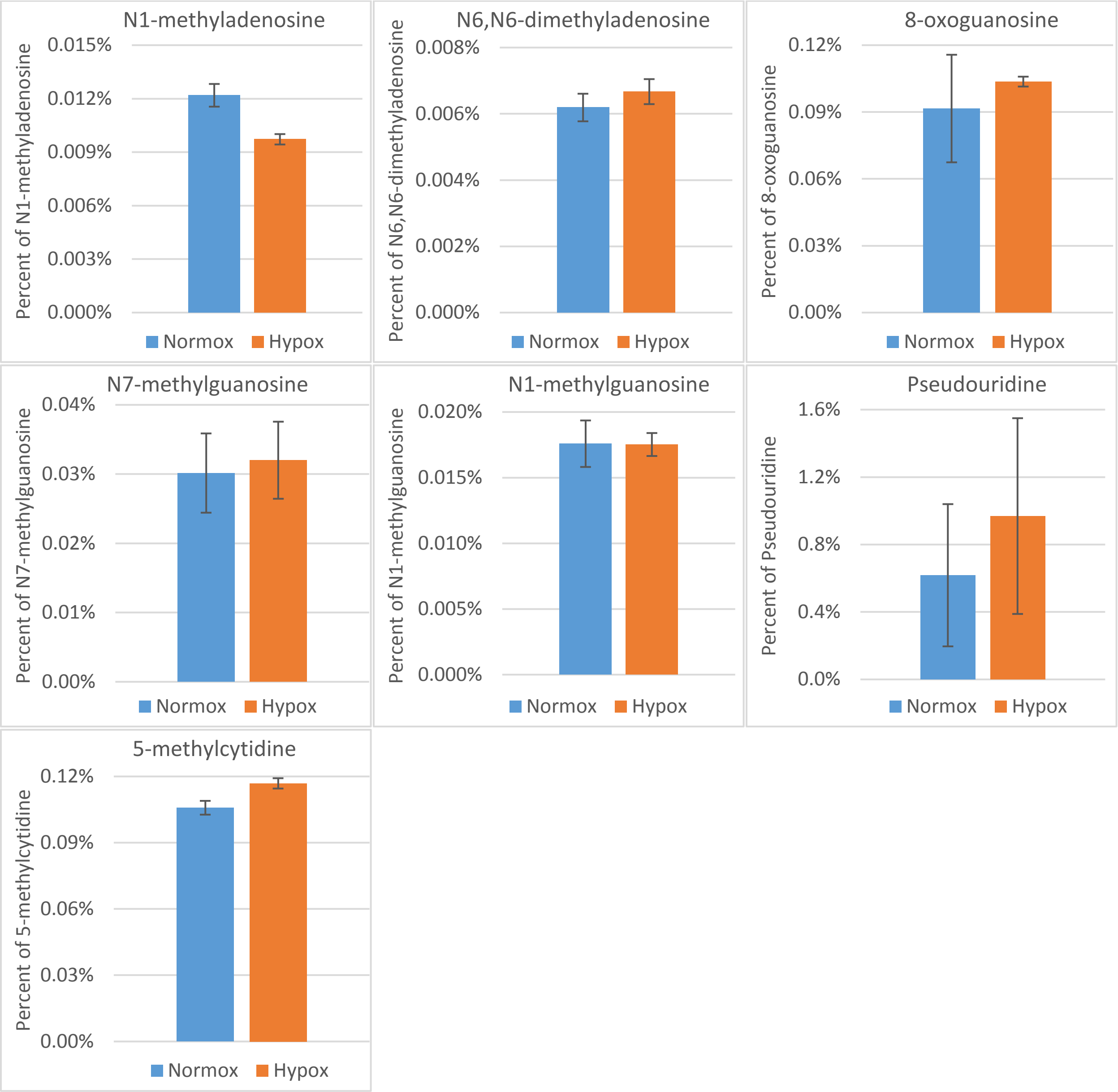

Supplemental Figure 2: UPLC-MS/MS of Poly A$^+$ RNA from HEK-293T cells cultured in Normoxic or 24 hours Hypoxic conditions. Values represent the amount of the modification divided by total parent levels (N of 2). *P ≤ 0.05 by Paired Student's t-test. Error bars represent Standard error of the mean (SEM).

Supplemental Figure 3 **UPLC-MS/MS of Total RNA**

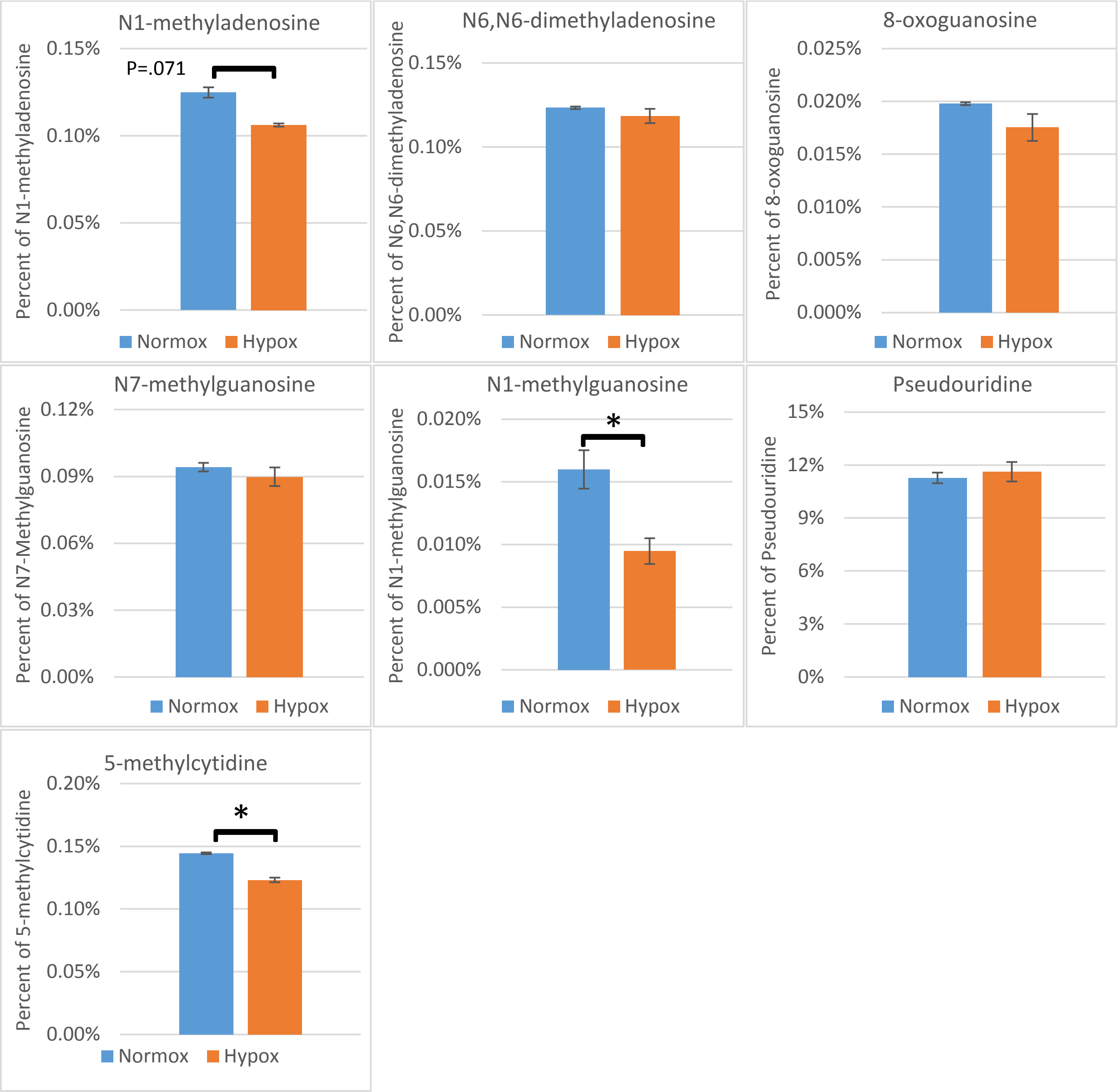

Supplemental Figure 3: UPLC-MS/MS of Total RNA from HEK-293T cells. Values represent the amount of the modification divided by total parent levels (N of 2). *P ≤ 0.05 by Paired Student's t-test. Error bars represent Standard error of the mean (SEM).

Supplemental Figure 4 **UPLC-MS/MS of Ribosomal RNA**

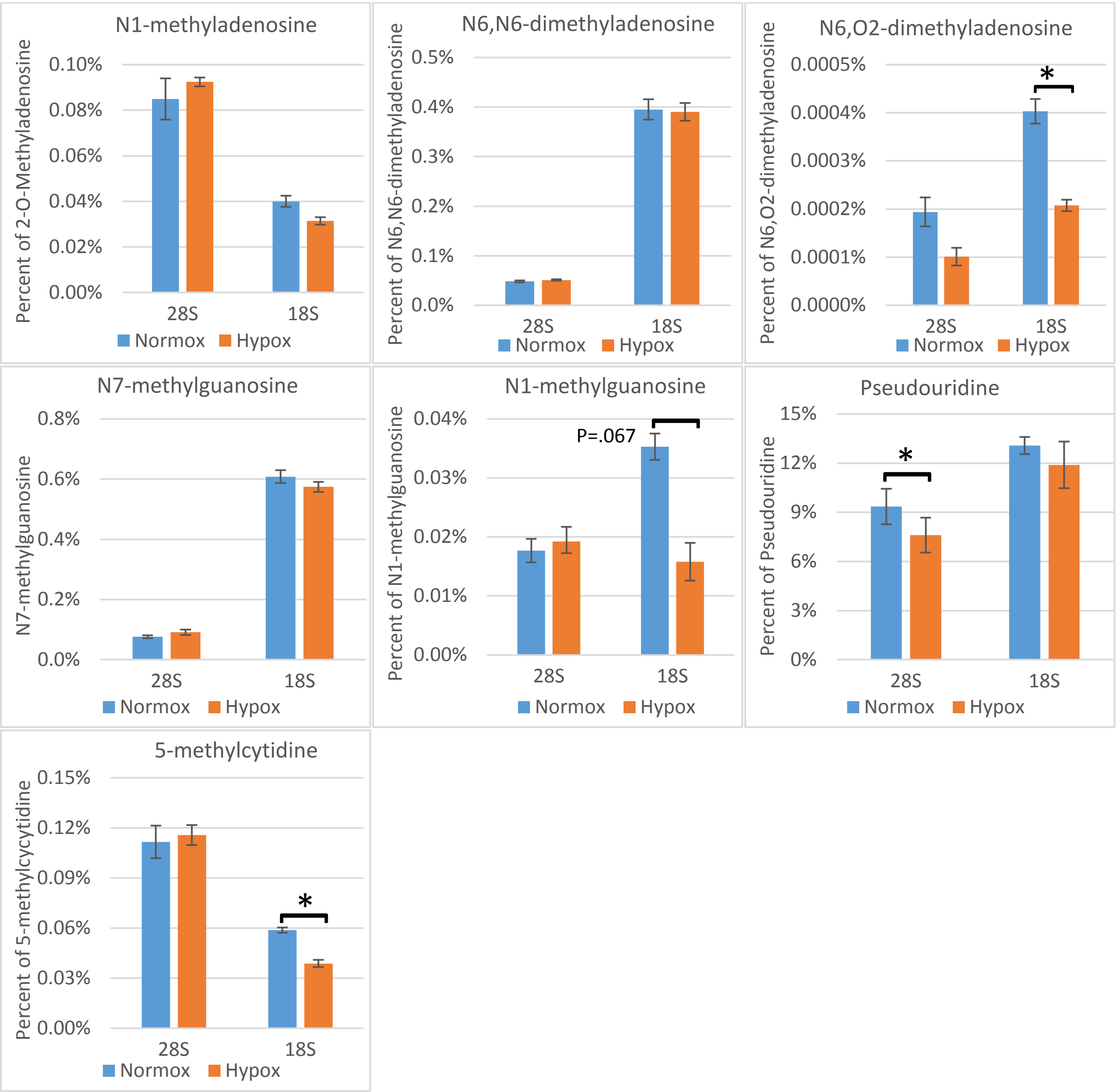

Supplemental Figure 4: UPLC-MS/MS of Ribosomal RNA from HEK-293T cells. Values represent the amount of the modification divided by total parent levels (N of 3). *P ≤ 0.05 by Paired Student's t-test. Error bars represent Standard error of the mean (SEM).

Supplemental Figure 5

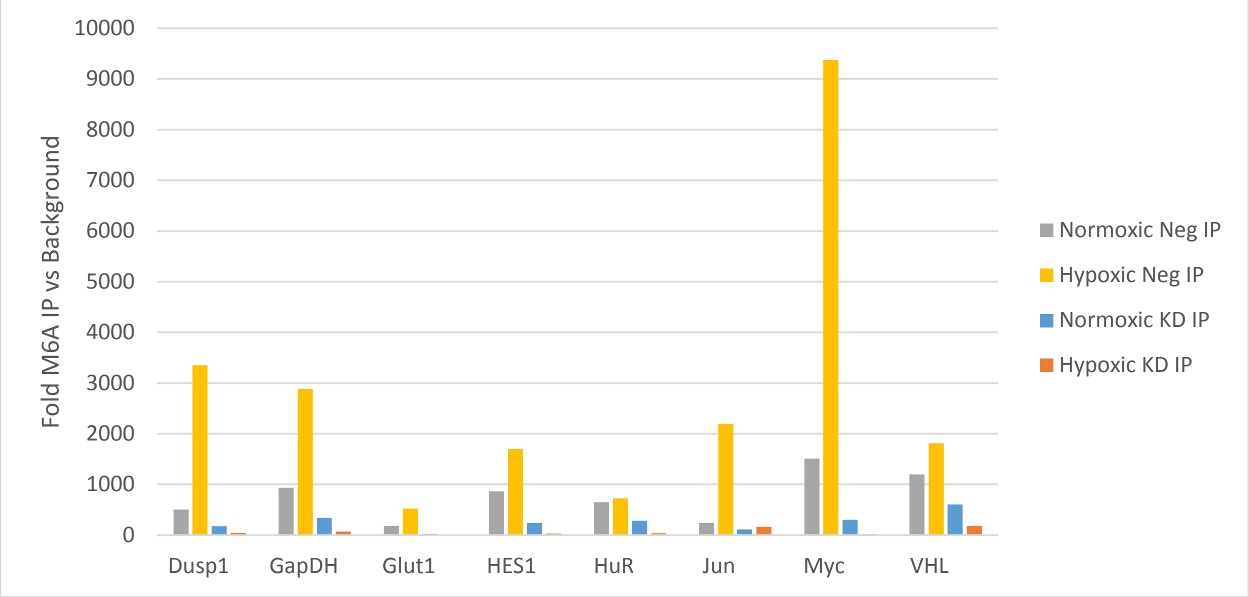

Supplemental Figure 5:

Transfection with METTL3 and METTL14 siRNAs decrease M6A levels.

HEK-293Ts transfected with METTL3 and 14 siRNAs (KD) or negative control siRNA (Neg) for 72 hours and 24 hours of normoxic or hypoxic exposure followed by MeRIP and qRT-PCR quantitation shows decreased M6A levels in mRNA targets after Mettl3/14 knockdown. (N of 1)

Supplemental Figure 6

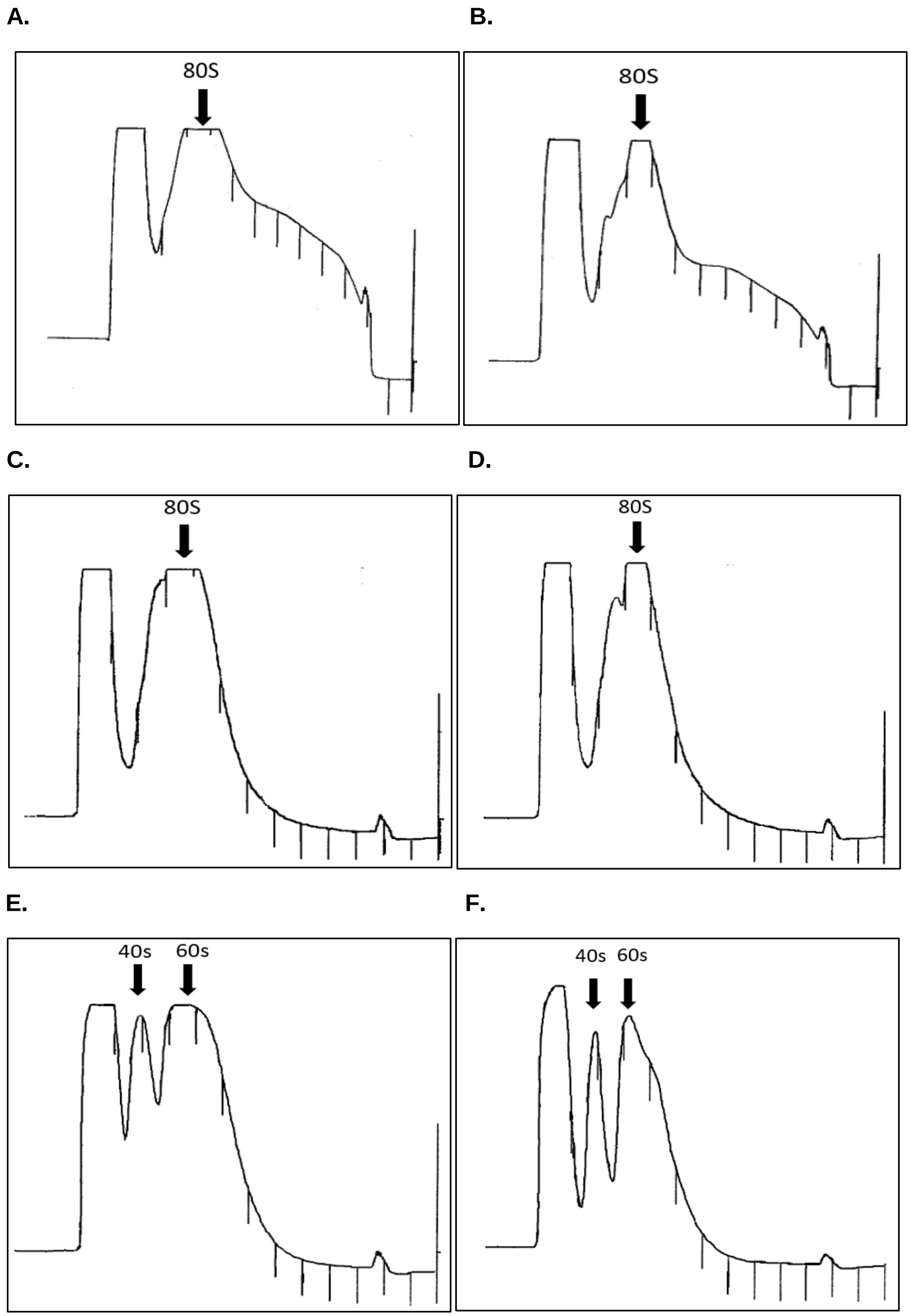

Supplemental Figure 6: Polysome Profile traces.

(A-D) Traces of Polysome Profiles from Figure 4. (A), Normoxia, (B), Normoxia M3/14 siRNA, (C), Hypoxia, (D) Hypoxia M3/14 siRNA. (E-F) Traces of Polysome Profiles from Supplemental Figure 6. (E) Normoxia, (F), Hypoxia. (Traces represent 1 of 3 experiments).

Supplemental Figure 7

A.

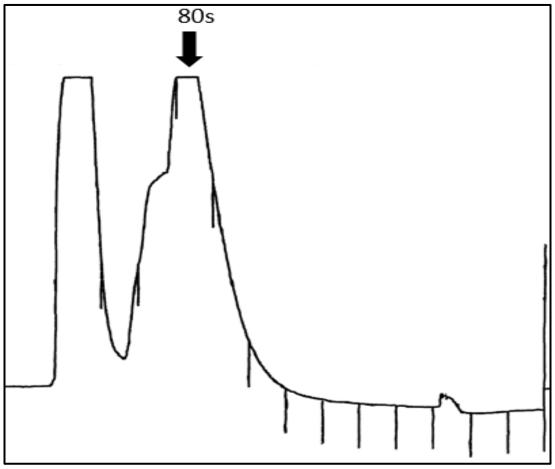

B.

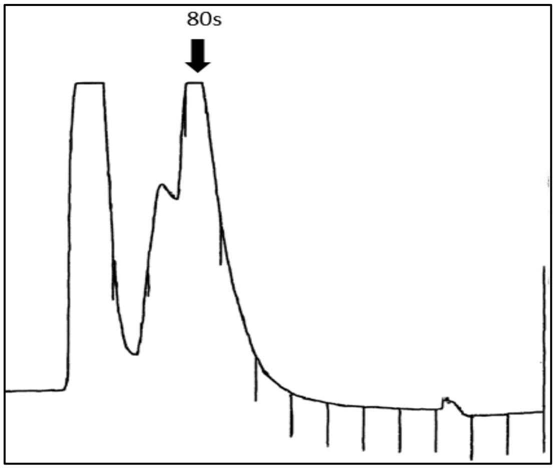

C.

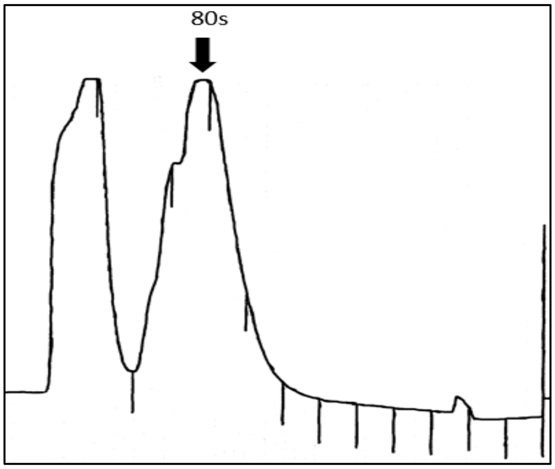

D.

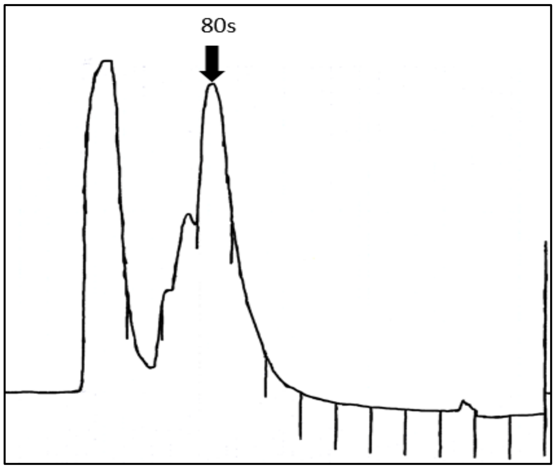

E.

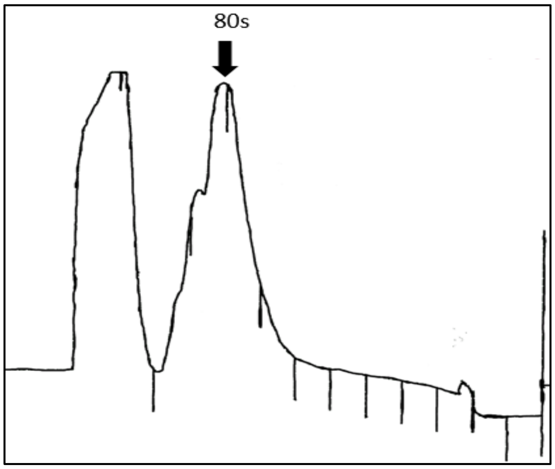

F.

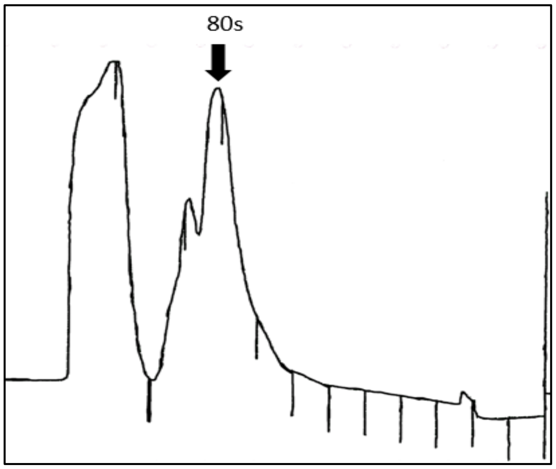

Supplemental Figure 7: Reoxygenation Polysome Profile traces.

(A-F) Traces of Polysome Profiles from Figure 5. (A), 30min reoxygenation negative siRNA, (B), 30min reoxygenation m3/14 siRNA, (C), 1hr reoxygenation negative siRNA, (D), 1hr reoxygenation m3/14 siRNA, (E), 4hr reoxygenation negative siRNA, (F),4hr reoxygenation m3/14 siRNA. (Traces represent 1 of 3 experiments).

Supplemental Figure 8

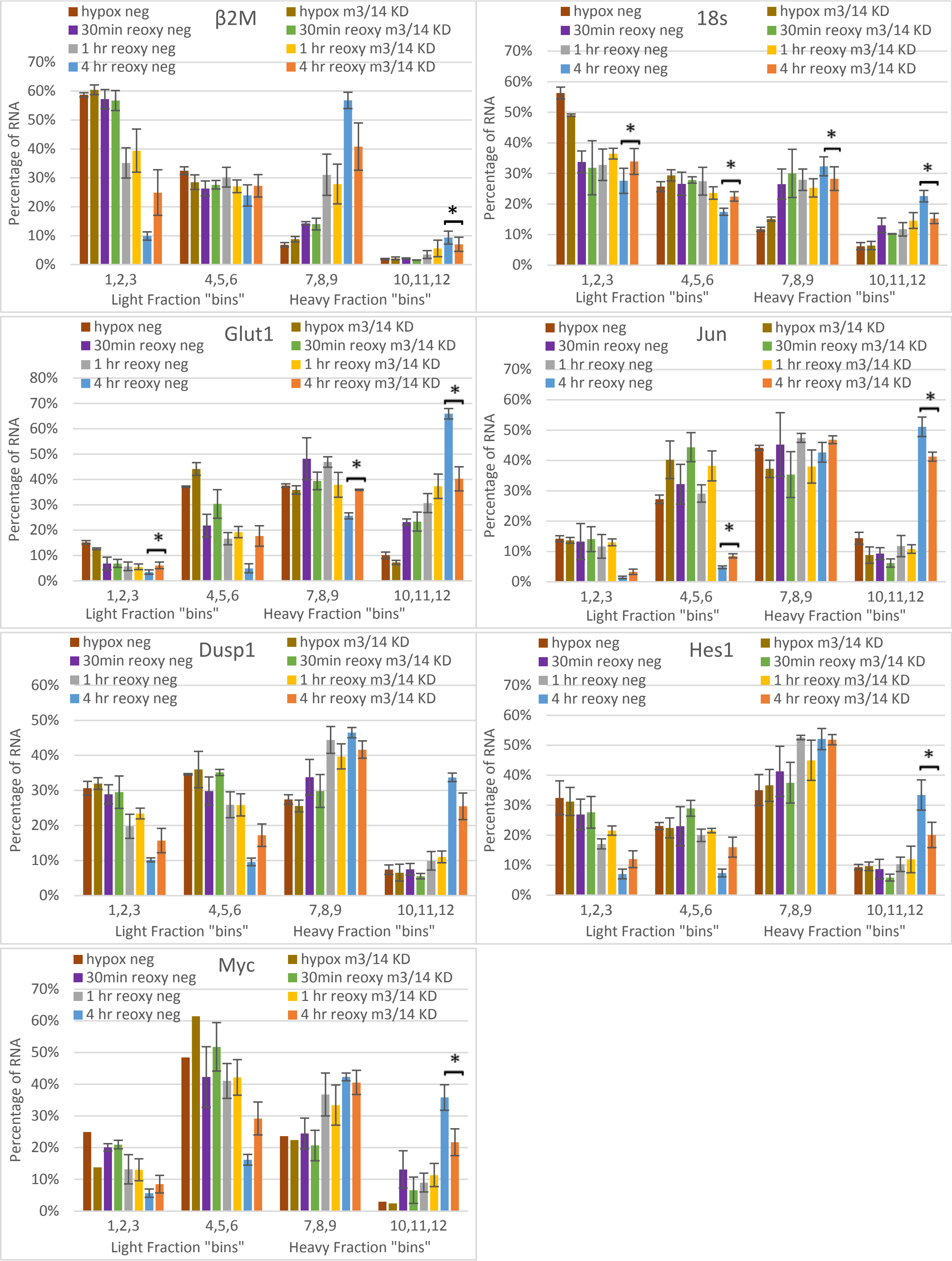

Supplemental Figure 8. Mettl3/14 Knockdown decreased the ability of messages to recover from hypoxic stress after 4 hours re-oxygenation.

HEK-293T cells harvested after 72 hours transfection with Mettl3/14 (M3/14 KD) or negative control (Neg) siRNA and 24 hours of hypoxic conditions and either 30 minutes, 1 hour or 4 hours of room level re-oxygenation recovery. Polysome Profiling of extracts separated by differential centrifugation followed by sucrose gradients. qRT-PCR analysis followed by binning into estimated non-polysome (1,2,3), light polysome (4,5,6), moderate polysome (7,8,9), and heavy polysome (10,11,12) bound fractions show percentage of individual mRNA in each bin. Error bars represent SEM of 3 experiments in the no re-oxygenation, 1 and 4 hour re-oxygenation experiments and SEM of 2 experiments in the 30 minute re-oxygenation experiment. N of 1 Myc hypoxia experiment is shown. *$P \leq 0.05$ by Paired Student’s t-test compared to binned negative control siRNA.

Supplemental Figure 9

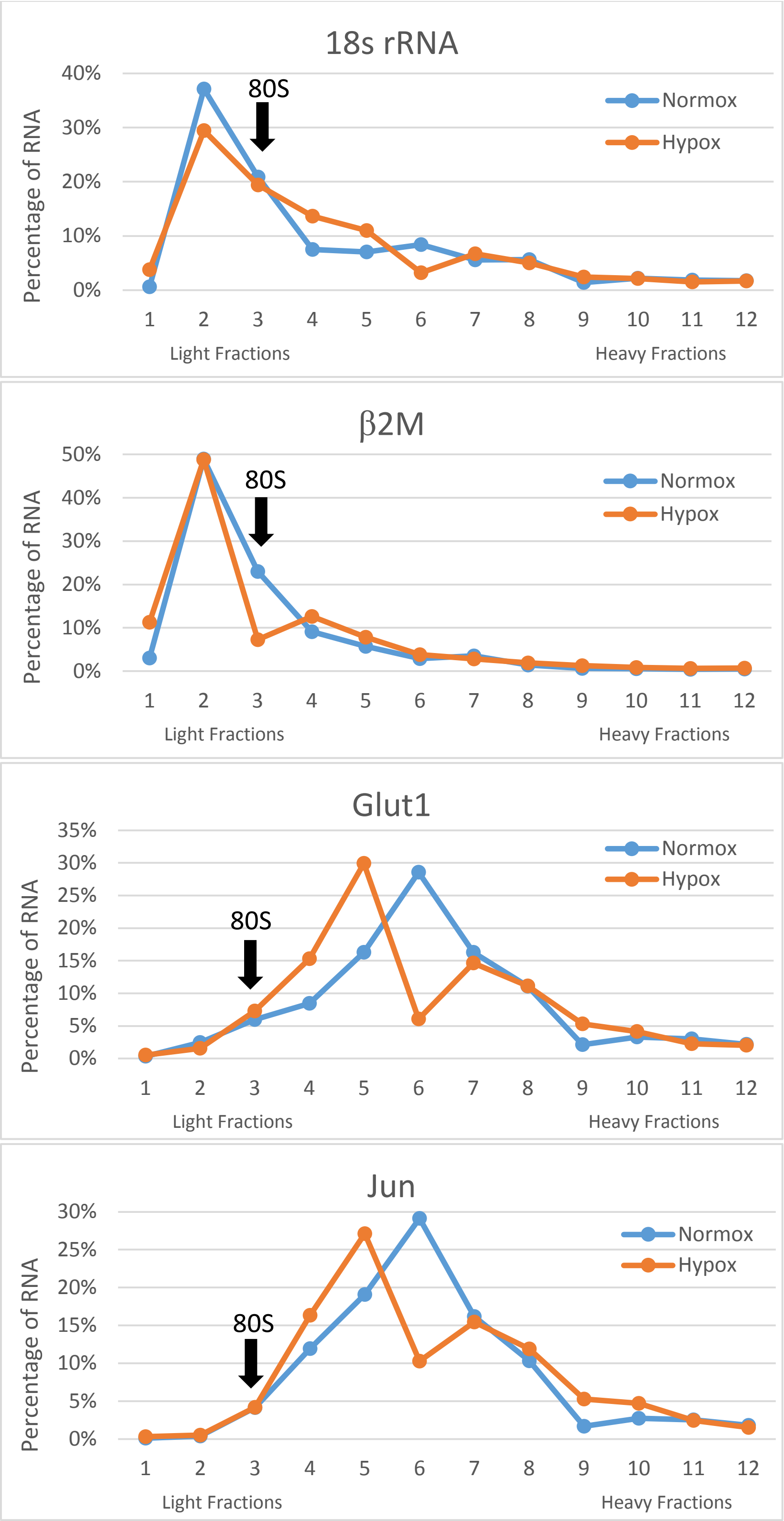

Supplemental Figure 9: EDTA-resistant RNP particles are observed for several hypoxia-modified mRNAs.

Polysome Profiling following addition of 25mM EDTA added to the lysis buffer. qRT-PCR analysis of the fractions shows percentage of the specific mRNA in each fraction. Fraction containing 80S peak is marked. Compare to Figure 3.

Supplemental Figure 10

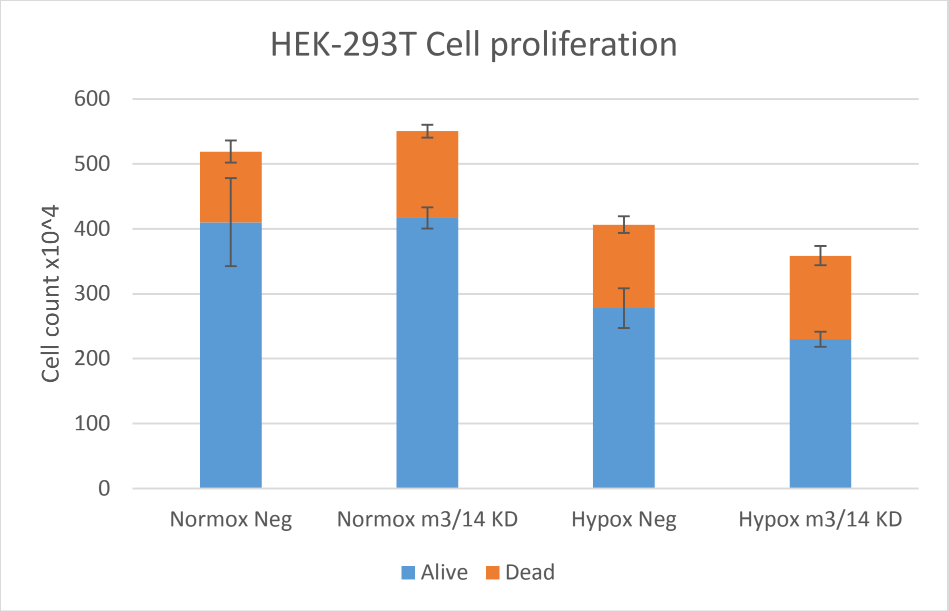

Supplemental Figure 10: Mettl3/14 Knockdown (m3/14 KD) had no significant effect on cell proliferation in HEK-293T cells grown in Normoxic (Normox) or 24 hour Hypoxic (Hypox) conditions when compared to negative control siRNA (Neg). (N of 3)

Supplemental Table 1

Primer List

Gene	Forward (5'-3')	Reverse (5'-3')
18s rRNA	CTGAGAAACGGCTACCACATC	GCCTCGAAAGAGTCCTGTATTG
28s rRNA	GGGTGGTAAACTCCATCTAAGG	GCCCTCTTGAACTCTCTCTTC
5.8s rRNA	CTCGTGCGTCGATGAAGAA	TCGAAGTGTCGATGATCAATGT
5s rRNA	CGTCTGATCTCGGAAGCTAAG	CCTACAGCACCCGGTATTC
β2M	AGATGTCTCGCTCCGTGGCCTTA	TGTCGGATGGATGAAACCCAGACA
Dusp1	CAACCACAAGGCAGACATCA	CAGTGGACAAACACCCTTCC
eEF1A1	CGGTCTCAGAACTGTTTGTTTC	AAACCAAAGTGGTCCACAAA
GapDH	AAGGTCGGAGTCAACGGATTTGGT	AGCCTTGACGGTGCCATGGAATTT
Glut1	TATCGTCAACACGGCCTTCACTGT	CACAAAGCCAAAGATGGCCACGAT
Hes1	GAAGGCGGACATTCTGGAAAT	GTCACCTCGTTCATGCACTC
HuR	CCTGTTCAGCAGCATTGGTGAAGT	TTCAGCGTGTTGATCGCTCTCTCT
Jun	TTCTATGACGATGCCCTCAAC	TCAGGGTCATGCTCTGTTTC
METTL3	AGCCTTCTGAACCAACAGTCC	CCGACCTCGAGAGCGAAAT
Myc	TCCTCGGATTCTCTGCTCTCCT	AGAAGGTGATCCAGACTCTGACCT
VHL	TCTCTCAATGTTGACGGACAGCCT	GGTCTTTCTGCACATTTGGGTGGT

Supplemental Table 2

siRNA's

siRNA	Target Sequence
siMETTL3	CTGCAAGTATGTTCACTATGA
siMETTL14	AAGGATGAGTTAATAGCTAAA
Negative Control #1 siRNA	Ambion cat. # AM4635

Supplemental Table 3

Antibodies

Antibody	Catalogue #	Vendor	WB Dilution	IP Amount
c-Jun	MA5-15119	Thermo Fisher	1/100	
c-Myc	NBP1-19671	Novus Biologicals	1/1000	
GAPDH (G-9)	sc-365062	Santa Cruz Biotechnology	1/100	
Glut1	PA5-16793	Thermo Fisher	1/250	
METTL14	HPA038002	Sigma Life Science	1/1000	
METTL3/MT-A70	A301-567A	Bethyl Laboratories	1/5000	
MKP-1 (c-19) (DUSP1)	sc-370	Santa Cruz Biotechnology	1/100	
N6-methyladenosine (m6a)	MABE1006	EMD Millipore		5 µg
